# Effect of Gastrointestinal Hemorrhage on Outcome After Endovascular Treatment in Acute Basilar Artery Occlusion

**DOI:** 10.3389/fneur.2022.809209

**Published:** 2022-04-28

**Authors:** Hao Zhang, Weipeng Dai, Weilin Kong, Zhenhui Duan, Zongjin Yun, Sheng Zhou, Jie Yang, Fengli Li, Wenjie Zi, Zhangbao Guo, Wenhua Liu

**Affiliations:** ^1^Department of Neurology, Affiliated Hangzhou First People's Hospital, Zhejiang University School of Medicine, Hangzhou, China; ^2^Department of Neurology, Jiangmen Central Hospital, Jiangmen, China; ^3^Department of Neurology, Xinqiao Hospital and The Second Affiliated Hospital, Army Medical University (Third Military Medical University), Chongqing, China; ^4^Department of Neurology, Wuhan No. 1 Hospital, Wuhan, China; ^5^Department of Neurology, Fuyang Hospital of Anhui Medical University, Fuyang, China; ^6^Department of Neurology, Renhuai People's Hospital, Guizhou, China

**Keywords:** gastrointestinal hemorrhage, gastrointestinal bleeding, endovascular treatment, acute basilar artery occlusion, posterior circulation

## Abstract

**Background and Purpose:**

Gastrointestinal hemorrhage (GIH) is associated with a poorer prognosis and a higher mortality rate after acute ischemic stroke (AIS), but its association with outcomes after endovascular treatment (EVT) remains unclear. This study aimed to assess the incidence, risk factors, and relationships among clinical outcomes of GIH after EVT in patients with acute basilar artery occlusion (BAO).

**Methods:**

Consecutive patients treated with EVT were identified from the EVT for Acute Basilar Artery Occlusion Study (BASILAR) registry. All enrolled patients were divided into GIH and non-GIH subgroups, and the independent predictors of GIH after EVT were explored. An ordinal logistic regression model was used to assess the association between GIH and primary outcome [distribution of modified Rankin scale (mRS)] at 90 days, while binary logistic regression models for other outcomes were also employed.

**Results:**

Among 647 patients with acute BAO, 114 (17.6%) patients experienced GIH after EVT. Higher glucose levels at admission, longer procedure time, and general anesthesia were the independent predictors of GIH after EVT, while protective factors include the posterior circulation-Acute Stroke Prognosis Early Computed Tomography Score (pc-ASPECTS) ≥ 5 and a history of hyperlipidemia. Compared with the non-GIH group, the GIH group was associated with a worse functional outcome [adjusted common odds ratio (OR), 2.12 (95% CI, 1.39–3.25)], lower rates of functional independence [adjusted OR,.47 (95% CI, 0.26–0.88)], a favorable outcome [adjusted OR, 0.41 (95% CI, 0.22–0.73)], and a higher risk of 90-day mortality [adjusted OR, 1.76 (95% CI, 1.08–2.85)].

**Conclusion:**

This study concluded that GIH is not uncommon after EVT in patients with acute BAO and is associated with worse functional disability and higher mortality.

## Introduction

Acute basilar artery occlusion (BAO) represents a devastating disease with a high rate of morbidity and mortality in patients with acute ischemic stroke (AIS) (1). Recent trials have shown mechanical thrombectomy to be a safe and effective treatment for acute vertebrobasilar artery occlusion (2–4). Successful recanalization of BAO after endovascular treatment (EVT) is an important prognostic factor for survival and good functional outcomes (5). The prognostic factors, such as stroke subtype, initial stroke severity, ischemic injury, and collateral status before thrombectomy, were identified as independent factors affecting the clinical outcome after EVT in patients with acute BAO (6).

Gastrointestinal hemorrhage (GIH) is a complication following AIS, with an incidence rate reported in a range of 1.24–8.6% (7, 8). GIH has been associated with a poorer outcome, death during the acute phase (9, 10), and the recurrence of stroke as recorded in the previous study (11). Many risk factors were reported to contribute to the occurrence of GIH after AIS, (12, 13) including low Glasgow Coma Scale (GSC) score, infection, posterior circulation infarction, peptic ulcer disease, and severe stroke. However, little literature was focused on the occurrence rate of GIH following EVT in patients with BAO, as well as the influence of GIH on clinical outcomes in interventional treated patients.

In this study, we aimed to determine the incidence and risk factors of GIH after EVT in patients with BAO. This study also aims to investigate the association between GIH and clinical outcomes among acute BAO patients receiving EVT.

## Materials and Methods

### Patient Selection

The EVT for Acute Basilar Artery Occlusion Study (BASILAR) was a nationwide prospective registry study of consecutive patients who presented with an acute, symptomatic, radiologically confirmed BAO in 47 comprehensive stroke centers in China and was registered on the Chinese Clinical Trial Registry (http://www.chictr.org.cn; ChiCTR1800014759). The study protocol was approved by the ethics committee of the local institutional review board of each center. We obtained written informed consent from patients or their legal authority representatives according to the Declaration of Helsinki. Patients receiving EVT in the BASILAR registry were included in this sub-analysis.

In general, the enrolled patients have to fulfill the following criteria: (1) 18 years or older; (2) presentation within 24 h of the estimated time of BAO; (3) BAO confirmed by CT-angiography, magnetic resonance angiography, or digital subtraction angiography; (4) initiation of intravenous recombinant tissue plasminogen activator (rt-PA) within 4.5 h or urokinase within 6 h of the estimated time of BAO if thrombolysis was available and approved; and (5) an ability to provide informed consent. All patients received standard medical treatment [e.g., intravenous thrombolysis (IVT) with rt-PA or urokinase, antiplatelet drugs, systematic anticoagulation, or combinations of these medical treatments] plus endovascular treatment, which included mechanical thrombectomy with stent retrievers and/or thromboaspiration, balloon angioplasty, stenting, intra-arterial thrombolysis, or the various combinations of these approaches. The exclusion criteria were the following: (1) the modified Rankin Scale (mRS) score ≥ 2 before admission; (2) cerebral hemorrhage before EVT; (3) an absence of 90-day outcomes as well as incomplete baseline imaging and time-metric data; (4) current pregnancy or lactation; and (5) a serious, advanced, or terminal illness.

### Baseline Characteristics

The baseline characteristics, including demographic data, history of medicine, laboratory measures, pretreatment, and posttreatment imaging findings, and treatment-related variables, were abstracted from the BASILAR registry. Stroke etiology was assessed according to the Trial of ORG 10172 in Acute Stroke Treatment criteria (TOAST) (14). Stroke severity was assessed with the National Institutes of Health Stroke Scale (NIHSS) score at admission (15). Pretreatment ischemic injury was evaluated with the posterior circulation Acute Stroke Prognosis Early Computed Tomography Score (pc-ASPECTS) (16). The extent of cerebral tissue reperfusion was assessed with the modified Thrombolysis in Cerebral Infarction (mTICI) score, and the grade of 2b or 3 was defined as successful reperfusion (17).

### Definition of GIH

Gastrointestinal hemorrhage was defined as any episode of fresh blood or coffee-ground emesis, hematemesis, melena, hematochezia, nausea and vomiting, diarrhea, and abdominal distention occurring within 72 h after endovascular treatment (18).

### Outcome Measures

The primary outcomes were evaluated with the mRS score at 90 days. The mRS score is a 7-level categorical scale and ranges from 0 (no symptoms) to 5 (severe disability) and 6 (death) (19). The mRS scores were determined by investigators who were blinded to the details of hospitalization. Other outcomes included length of hospital stay, in-hospital mortality, 90-day favorable outcome (defined as mRS 0–3), 90-day functional independence (mRS 0-2), and all-cause mortality within 90 days. The median length of hospital stay (LOS) in the study was 12 days. For analyses, the LOS was dichotomized as “short LOS” (LOS ≤ 12 d) and “long LOS” (LOS > 12 d).

### Statistical Analysis

All statistical analyses were performed using SPSS 23 version (IBM Corp, Armonk, NY, USA). A two-tailed *P*-value < 0.05 was considered statistically significant. Continuous variables were analyzed by the Mann–Whitney U test, and categorical variables were analyzed by the χ2 or Fisher exact tests. The factors independently predicting the risk of GIH after EVT were explored using stepwise multivariate logistic regression analysis, adjusting for confounders with *P* < 0.1 in univariate analysis or with clinical relevance.

To assess the effect of GIH on the primary outcome, a proportional odds model was performed for a shift analysis toward 1 category of functional deterioration. The adjusted common odds ratios (cOR) were reported with 95% CI. The length of stay, in-hospital mortality, dichotomized scores of mRS at 90 days, and mortality within 90 days were analyzed using binary logistic regression, with the OR as the effect measure. Each multivariate model had the following confounders: age, history of diabetes, baseline NIHSS, baseline pc-ASPECTS, successful recanalization, and location of the occlusion. In a sensitivity analysis, the forest plot was used to represent the relationship between GIH and primary outcome in each subgroup. The assessment of effect heterogeneity of GIH was also performed with the inclusion of interaction terms.

## Results

### Baseline Characteristics

A total of 647 patients treated with EVT were enrolled in the analyses. Among them, the median age was 64 years (IQR, 56–73), and 483 patients (74.7%) were men. A total of 522 patients (80.7%) achieved successful recanalization, while 114 patients (17.6%) were observed with symptoms of GIH within 72 h after EVT.

Baseline characteristics and treatment features of the patients with BAO based on GIH are shown in Table 1. Compared with patients in the non-GIH group, patients in the GIH group had higher levels of glucose at admission (*P* = 0.011), higher rates of general anesthesia (*P* = 0.004), and rescue treatment including balloon angioplasty and/or stenting (*P* = 0.014). The time from puncture to recanalization (*P* < 0.001) and time from onset to recanalization (*P* = 0.026) were also longer in patients in the GIH group than those in the non-GIH group.

**Table 1:** Baseline characteristics and treatment features of patients with basilar artery occlusion (BAO), having with and without gastrointestinal hemorrhage (GIH).

**Characteristics**	**All patients (*n* = 647)**	**GIH (*n* = 114)**	**Non-GIH (*n* = 533)**	***P*** **-value**
Age (yrs), median (IQR)	64 (56–73)	65 (57–71)	64 (56–73)	0.689
Sex (male), *n* (%)	483 (74.7)	91 (79.8)	392 (73.5)	0.162
Baseline NIHSS score, median (IQR)	27 (17–33)	25 (18–33)	27 (16–34)	0.721
Baseline pc-ASPECTS, median (IQR)^*^	8 (7–9)	8 (6–9)	8 (7–9)	0.086
BATMAN, median (IQR)^†^	4 (2–6)	4 (2–5)	4 (2–6)	0.875
**Vascular risk factor**, ***n*** **(%)**				
Smoking	235 (36.3)	44 (38.6)	191 (35.8)	0.578
Hypertension	451 (69.7)	82 (71.9)	369 (69.2)	0.569
Hyperlipidemia	214 (33.1)	31 (27.2)	183 (34.3)	0.141
Diabetes mellitus	149 (23)	31 (27.2)	118 (22.1)	0.245
Drinking	141 (21.8)	26 (22.8)	115 (21.6)	0.773
**Medical history**, ***n*** **(%)**				
Atrial fibrillation	136 (21)	20 (17.5)	116 (21.8)	0.316
Coronary artery disease	105 (16.2)	19 (16.7)	86 (16.1)	0.889
Cerebral infarction	140 (21.6)	24 (21.1)	116 (21.8)	0.867
Intracerebral hemorrhage	12 (1.9)	1 (0.9)	11 (2.1)	0.346^§^
Pre-admission GIH	5 (0.8)	1 (0.9)	4 (0.8)	0.622^§^
Pre-admission antiplatelet	169 (26.2)	37 (32.5)	132 (24.9)	0.145^§^
Pre-admission anticoagulation	13 (2)	1 (0.9)	12 (2.3)	0.298
**Laboratory measures, median (IQR)**				
Glucose on admission	7.4 (6.1–9.7)	7.9 (6.9–11.1)	7.3 (6.0–9.4)	0.011
Platelet	211 (174–252)	213 (177–248)	211 (171–254)	0.779
Thrombosis time	17.2 (15–19.3)	16.9 (14.9–19.4)	17.2 (15–19.3)	0.642
Prothrombin time	12 (11.2–13.2)	12 (11–13.3)	11.9 (11.3–13.1)	0.705
Activated partial thromboplastin time	28.5 (25–33)	28.2 (24.8–33.4)	28.6 (25.1–33)	0.955
INR	1.03 (0.97–1.1)	1.04 (0.95–1.1)	1.02 (0.97–1.1)	0.791
D-Dimer	702 (253–2115)	720 (190–1865)	700 (270–2310)	0.604
Cause of stroke, *n* (%)				0.435
Large artery atherosclerosis	418 (64.6)	78 (68.4)	340 (63.8)	
Cardioembolism	173 (26.7)	25 (21.9)	148 (27.8)	
Other causes	56 (8.7)	11 (9.6)	45 (8.4)	
Occlusion sites, *n* (%)				0.267
Distal basilar artery	222 (34.3)	31 (27.2)	191 (35.8)	
Middle basilar artery	195 (30.1)	35 (30.7)	160 (30)	
Proximal basilar artery	107 (16.5)	21 (18.4)	86 (16.1)	
Vertebral artery-V4	123 (19)	27 (23.7)	96 (18)	
Intravenous thrombolysis, *n* (%)	119 (18.4)	22 (19.3)	97 (18.2)	0.867
Onset-treatment time, median (IQR), min	246 (132–390)	268.5 (131–485.3)	241 (132–378)	0.154
Puncture-recanalization time, median (IQR), min^‡^	105 (71–151)	130 (83–180)	101 (70–140)	<0.001
Onset-Recanalization time, median (IQR), min	441 (328–626)	459 (359–766)	435 (323–608)	0.026
General anesthesia, *n* (%)	257 (39.7)	59 (51.8)	198 (37.1)	0.004
Balloon angioplasty or stenting, *n* (%)	306 (47.5)	66 (57.9)	240 (45.3)	0.014
Successful recanalization, *n* (%)	522 (80.7)	86 (75.4)	436 (81.8)	0.118

* *Data were missing for 1 patient in the GIH cohort and 3 patients in the Non-GIH cohort*.

† *Data were missing for 1 patient in the Non-GIH cohort*.

‡ *Data were missing for 1 patient in the GIH cohort and 2 patients in the Non-GIH cohort*.

§ *Fisher exact test*.

*BAO, basilar artery occlusion; GIH, gastrointestinal hemorrhage; IQR, interquartile; NIHSS, National Institutes of Health Stroke Scale; pc-ASPECTS, posterior circulation-Alberta Stroke Program Early CT Score; BATMAN, basilar artery on Tomography Angiography*.

### Risk Factors for GIH

Table 2 showed the multivariate logistics regression analysis results for potential predictors of GIH following EVT. In adjusted analysis, the baseline pc-ASPECTS ≥ 5 scores [adjusted OR,.24 (95% CI, 0.08–0.72)] and history of hyperlipidemia [adjusted OR, 0.57 (95% CI, 0.34– 0.96)] were associated with a lower risk of GIH after EVT. Higher glucose levels at admission [adjusted OR, 1.08 (with 95% CI, 1.01–1.16)], longer procedure time [adjusted OR 1.08 (95% CI, 1.03–1.14)], and the effect of general anesthesia [adjusted OR, 2.05 (95% CI, 1.28–3.29)] were the independent predictors for GIH after EVT. Age, intravenous thrombolysis, and successful recanalization were not significantly associated with GIH after EVT.

**Table 2:** Multivariate analysis: predictors of GIH following endovascular treatment.

**Variables**	**Unadjusted OR (95% CI)**	***P*** **value**	**Adjusted OR (95% CI)**	***P*** **-value**
Age	1.00 (0.98–1.02)	0.769		
Sex	1.42 (0.87–2.34)	0.163		
Baseline NIHSS	1.00 (0.98–1.02)	0.847		
Baseline pc-ASPECTS ≥ 5	0.41 (0.17–0.98)	0.045	0.24 (0.08–0.72)	0.011
Hyperlipidemia	0.71 (0.46–1.12)	0.143	0.57 (0.34–0.96)	0.034
Glucose on admission	1.08 (1.01–1.15)	0.025	1.08 (1.01–1.16)	0.025
Intravenous thrombolysis	1.08 (0.64–1.80)	0.783		
Puncture-recanalization time	1.08 (1.04–1.13)	<0.001	1.08 (1.03–1.14)	0.003
General anesthesia	1.89 (1.25–2.86)	0.002	2.05 (1.28–3.29)	0.003
Balloon angioplasty or Stenting	1.66 (1.10–2.50)	0.015		
Successful recanalization	0.68 (0.42–1.10)	0.120		
Occlusion sites				
Distal basilar artery	Reference	NA		
Middle basilar artery	1.35 (0.80–2.28)	0.267		
Proximal basilar artery	1.51 (0.82–2.77)	0.189		
Vertebral artery-V4	1.73 (1.00–3.07)	0.059		

*GIH, gastrointestinal hemorrhage; NIHSS, National Institutes of Health Stroke Scale; pc-ASPECTS, Posterior Circulation Acute Stroke Prognosis Early Computed Tomography Score*.

### GIH and Clinical Outcomes

**Figure 1:**
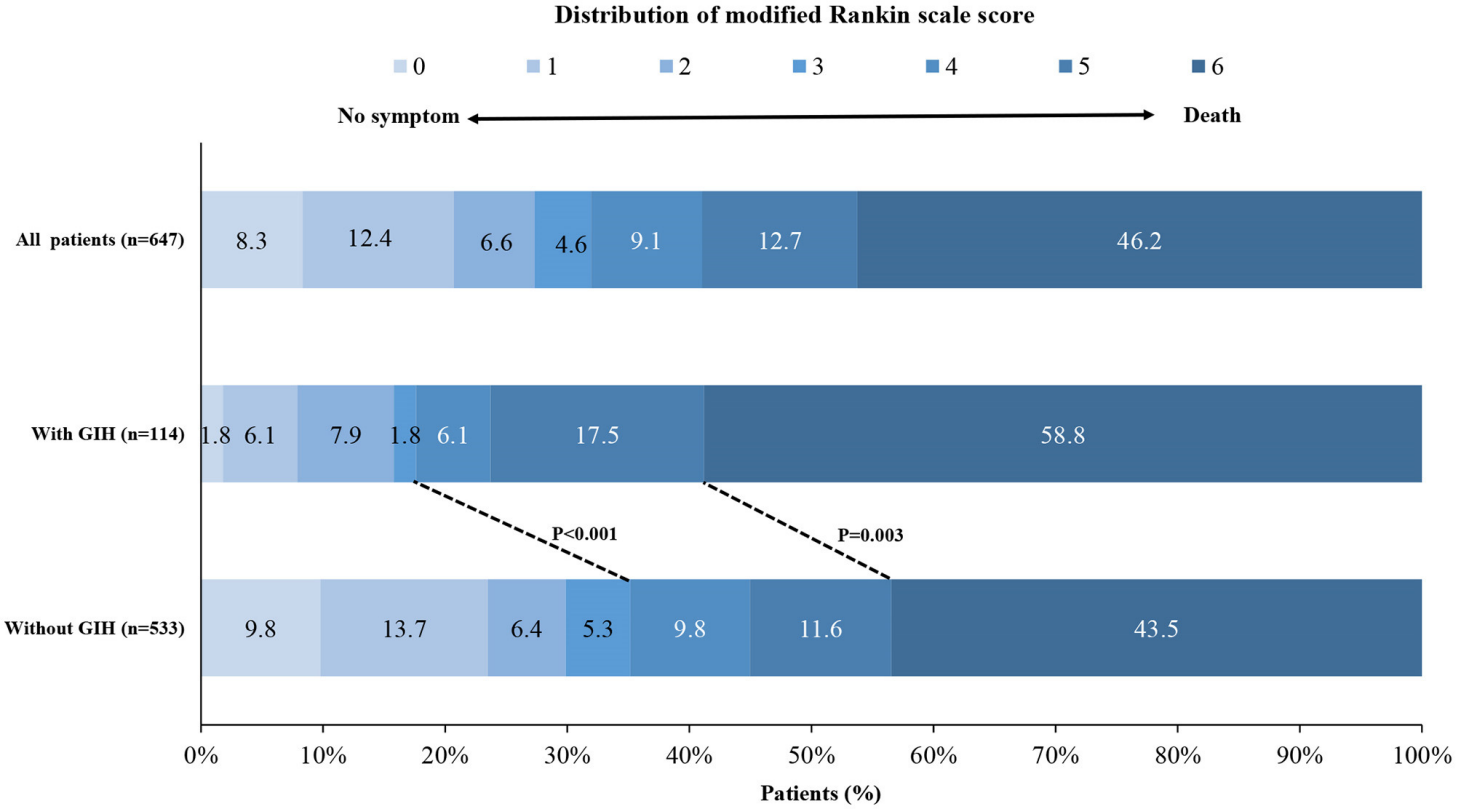
Distribution of the modified Rankin scale score at 90 days. Shown is the distribution of the modified Rankin scale (mRS) score among patients in the gastrointestinal hemorrhage (GIH) cohort and the non-GIH cohort. GIH indicates gastrointestinal hemorrhage.

The median 90-day mRS was 6 (IQR, 5–6) in patients with GIH and 5 (IQR, 2–6) in patients with non-GIH (Table 3 and Figure 1). The patients with GIH seemed to have higher rates of LOS > 12 d and in-hospital mortality than patients with no GIH, but the differences were not statistically significant. In patients with GIH, the rates of favorable outcomes and functional independence at 90 days were lower, but the rate of mortality within 90 days was higher compared to patients with non-GIH (Table 3).

**Table 3:** The effects of GIH on clinical outcomes after EVT in patients with acute basilar artery occlusion.

**Characteristic**	**All** **(*n* = 647)**	**GIH** **(*n* = 114)**	**Non-GIH (*n* = 533)**	**Unadjusted values (95% CI)**	***P*** **-value**	**Adjusted values (95% CI)**	***P*** **-value**
**Primary outcome**							
90d mRS, median (IQR)	5 (2–6)	6 (5–6)	5 (2–6)	2.13 (1.45–3.13)^*^	<0.001	2.12 (1.39–3.25)^*^	0.001
**Secondary outcomes, n/total** ***n*** **(%)**							
Length of stay >12d	295 (45.7)	58 (50.9)	237 (44.6)	1.29 (0.86–1.93)^†^	0.225	1.36 (0.89–2.10)^†^	0.155
In–hospital mortality	151 (23.5)	32 (28.1)	119 (22.5)	1.34 (0.85–1.12)^†^	0.208	1.19 (0.72–1.96)^†^	0.503
Mortality within 90d	299 (46.2)	67 (58.8)	232 (43.5)	1.85 (1.23–2.79)^†^	0.003	1.76 (1.08–2.85)^†^	0.022
90d mRS 0–2	177 (27.4)	18 (15.8)	159 (29.8)	0.44 (0.26–0.75)^†^	0.003	0.47 (0.26–0.88)^†^	0.018
90d mRS 0–3	207 (32.0)	20 (17.5)	187 (35.1)	0.39 (0.24–0.66)^†^	<0.001	0.41 (0.22–0.73)^†^	0.003

* *The common odds ratios were obtained using ordinal logistic regression*.

† *The odds ratios were estimated using binary logistic regression models*.

*Adjusted confounders included age, history of diabetes, baseline NIHSS, baseline pc-ASPECTS, successful recanalization, and occlusion site*.

*GIH, gastrointestinal hemorrhage; IQR, interquartile; CI, confidence interval; EVT, endovascular treatment; NIHSS, National Institutes of Health Stroke Scale; pc-ASPECTS, Posterior Circulation Acute Stroke Prognosis Early Computed Tomography Score*.

After adjustment for confounders, there was a shift toward worse outcome across the mRS categories with GIH, and the adjusted common OR was 2.12 (95% CI, 1.39–3.25, *P* < 0.001; Table 3). The adjusted ORs of GIH for a functional independence outcome, a favorable outcome, and 90-day mortality were.47 (95% CI, 0.26–0.88), 0.41 (95% CI, 0.22–0.73), and 1.76 (95% CI, 1.08–2.85), respectively.

### Subgroup Analysis

**Figure 2:**
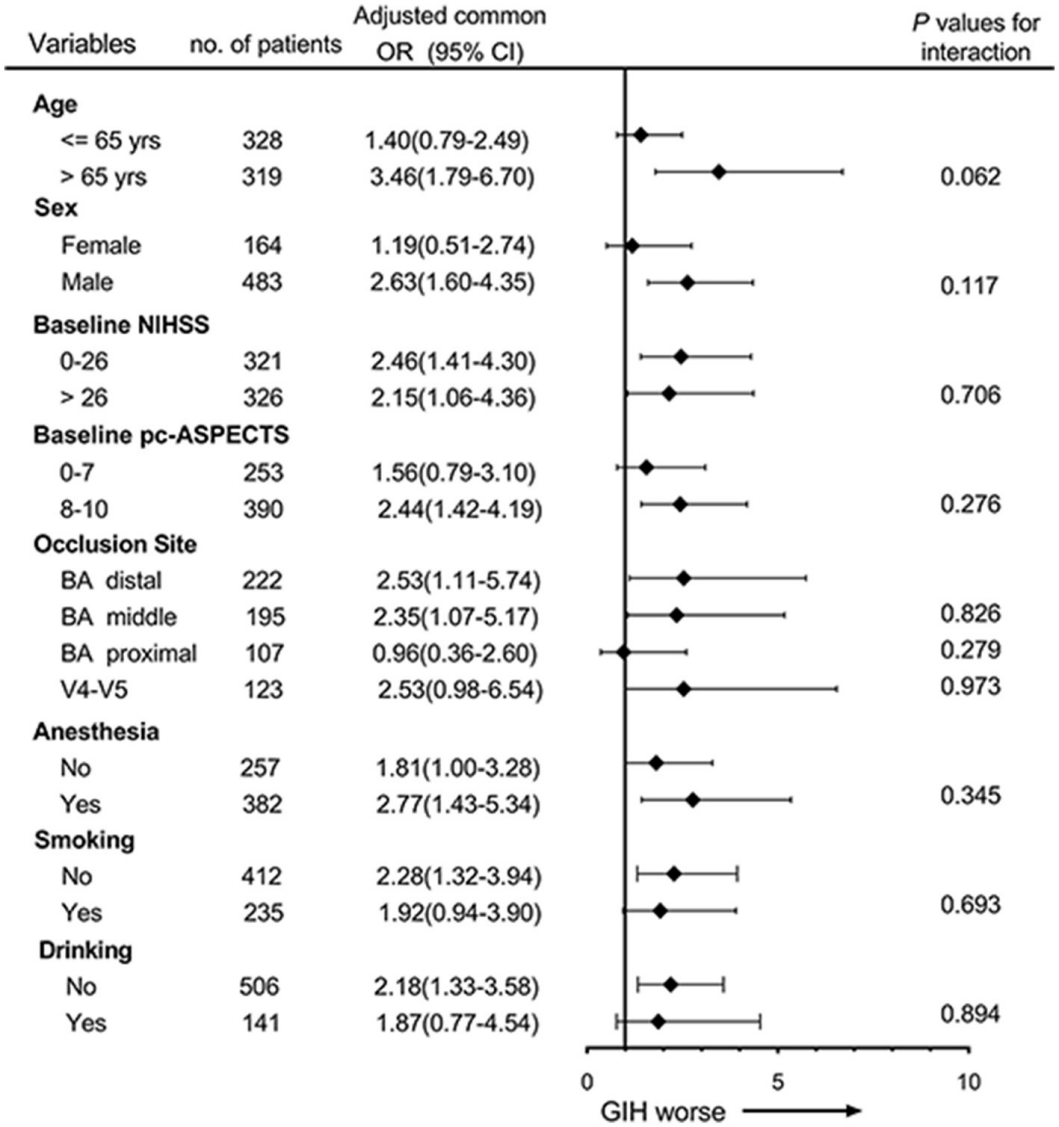
Subgroup analyses of primary outcome by ordinal logistic regression. The forest plot shows the effects of GIH on the primary outcome (common odds ratios indicating the addition of 1 point on the modified Rankin scale) at 90 days across the prespecified subgroups. Adjusting confounders included age, history of diabetes, baseline National Institutes of Health Stroke Scale (NIHSS), baseline posterior circulation Acute Stroke Prognosis Early Computed Tomography Score (pc-ASPECTS), occlusion site, and successful recanalization. The thresholds for age, NIHSS, and pc-ASPECTS were chosen at the median.

The forest plot shows that there was no significant heterogeneity of GIH on the primary outcome across the subgroups according to the interaction analysis (Figure 2). However, the harmful effects of GIH on the primary outcome seemed to be mild in patients aged ≤65, in women, and patients with proximal occlusion of the basilar artery.

## Discussion

This study first determined that GIH was related to an increased negative clinical functional outcome in patients with BAO who underwent EVT. The risk of poor outcomes increased approximately 1.13-folds in patients with GIH.

Up to now, few works of literature have reported the occurrence of GIH in patients with ischemic stroke with EVT. Previous studies showed the frequency of Asian GIH to be ranging from 1.4 (7) to 8.6% (8) in patients with AIS. Our study showed that the ratio of GIH followed by EVT was 17.6% (114/647) in patients with acute BAO, which suggested higher morbidity than the traditional standard medical treatment patients with anterior circulation stroke. The causes of GIH after EVT may have resulted from stress- or medication-related mucosal injury and ulcer. During the acute stroke stage, the interruption of the axis that connects the central nervous and the digestive system, also called the brain-gut axis, may increase the risk of gastrointestinal mucosal injury. Therefore, large area cerebral ischemic stroke and posterior circulation ischemia possibly have a bigger risk for GIH. The disorders of the autonomic nervous system pathway descending from the hypothalamus *via* the mesencephalon, the pons, and the medulla to the spinal cord may account for the association between posterior circulation ischemia and GIH. However, the mechanisms on how the GIH affects neurological outcomes remain unknown. One widely accepted hypothesis currently is the adverse effect of hypoperfusion (20). Once GIH occurs after EVT, even though it is mild, the patients must discontinue the treatment of anti-platelet drugs, which leads to a prothrombotic status. Fasting and gastrointestinal decompression also produce hemodynamic insufficiency. All these factors jointly result in a poor clinical neurological outcome.

According to the summary of previous studies, the use of alteplase can also cause other forms of life-threatening bleeding, most commonly gastrointestinal bleeding. A total of 119 patients were treated with IVT in this study. Among the group of patients in the GIH group, 22 (19.3%) were treated with intravenous alteplase (0.9 mg/Kg weight, maximum dose 90 mg), while a total of 97 patients (18.2%) in the non-GIH group were treated with intravenous alteplase within 4.5 h of onset based on the Chinese guidelines and the guidelines for the management of AIS. However, there was found no difference in the rate of intravenous alteplase between the two groups. Similarly, one previous research in the United States suggested an inconsistent result that patients with AIS who received thrombolytic therapy had a lower rate of GIH. Perhaps because of the limited number of people, (9) our study showed that general anesthesia was a risk factor for GIH following EVT. One meta-analysis showed that patients with EVT who received general anesthesia would have more severe complications and suffer worse outcomes than patients (21) who received local anesthesia, but no related works of literature were recorded about the effect of general anesthesia on GIH after EVT. The mechanism may result from the hemodynamic change during general anesthesia and consequential reperfusion injury (22). Balloon angioplasty or stenting was a risk in univariate logistic regression but was not associated with GIH in multivariate analysis. In previous studies, atrial fibrillation, oral anticoagulant use, brain herniation, male sex, infection, and posterior circulation infarction had been shown to increase the risk of GIH (8, 10, 11). In addition to this finding, the current study found that puncture to recanalization time, glucose level on admission, pc-ASPECTS score, general anesthesia, and number of AICA was the related factor for GIH. There was a publication that researched the impact of GIH history on the outcomes after percutaneous coronary intervention in patients with cardiovascular disease and found that GIH history increased the in-hospital bleeding complications (23), which was different from our results. Quick recanalization during EVT can shorten the onset-to-reperfusion time, which could cut down the level of oxygen free radicals in the body and relieve gut inflammatory and immune responses. The improvement of gastrointestinal environment imbalance, such as gut microbiota dysbiosis and poststroke leaky gut, decreases the rate of GIH in return (24).

It remains unclear whether there will be advantages to treating GI bleeding. The use of a proton pump inhibitor (PPI) can reduce the GI bleeding risk in the acute stroke stage, which will help improve the tolerance of antiplatelet or anticoagulant agents and prevent hemodynamic instability or brain hypoperfusion. Previous studies indicated that in-hospital GI bleeding increased the rate of respiratory complications (e.g., pneumonia) and stroke recurrence (25), which means that treating GI bleeding may decrease the prevalence of respiratory complications and recurrent stroke. Subsequently, the clinical outcomes and the life quality of patients would be improved. Furthermore, it will provide evidence of what happened to patients with GIH following EVT and present possible recommendations for a better guide for interventional neurologists. Further studies are required to explore the mechanisms for their associations.

The strengths of our study are that it contained a larger sample size compared with other studies and it is a multicenter study that eliminated the bias originating from single-center studies, which is common in previous works of literature. Moreover, this is the first study to our knowledge that described the GI bleeding following EVT. However, some limitations should not be ignored. (1) In this manuscript, GIH was defined as any episode of fresh blood or coffee-ground emesis, hematemesis, melena, hematochezia, nausea and vomiting, diarrhea, and abdominal distention occurring within 72 h after endovascular treatment. We attempted to further analyze the hemodynamic significance of GIH. However, the hemodynamic significance of GIH could not be effectively analyzed due to the small amount of blood loss and complex hemodynamic detection methods. (2) Notably, the symptoms of melena should rule out the influence of some food and medicine, and hematochezia might be caused by anorectal diseases, such as hemorrhoids and anal fissures. Nausea and vomiting were also not very specific symptoms of GIH. The lack of a continuous record of these symptoms could lead to false positives. Moreover, this study might have exaggerated the incidence of GIH in patients without using the endoscopy, since AIS is often considered a relative contraindication to endoscopy. (3) There was no authoritative definition of time for GIH after EVT, which means that we could not eliminate the influence of antiplatelet and anticoagulant drugs. (4) Finally, the dose of the antiplatelet drug and the use of the proton-pump inhibitor after EVT were not recorded in this study, which may affect the GIH event.

## Conclusion

Gastrointestinal hemorrhage influences the outcomes in the AIS after EVT. Reducing the relevant risk factors and the application of consuming prophylactic medications should be taken into consideration to minimize the occurrence of GIH.

## Data Availability Statement

The raw data supporting the conclusions of this article will be made available by the authors, without undue reservation.

## Ethics Statement

The studies involving human participants were reviewed and approved by Ethics Committee of the Xinqiao Hospital, Army Medical University. The patients/participants provided their written informed consent to participate in this study.

## Author Contributions

HZ, WD, and WK interpreted the data and drafted the manuscript. ZG and WL contributed to the conception and design of the study. ZD and ZY performed the statistical analyses. Acquisition, analysis, and interpretation of data were performed by SZ. JY, FL, and WZ provided technical and material support and also made critical revisions to the manuscript. All authors contributed to the article and approved the submitted version.

## Funding

This work was supported by the Medical and Health Project of Wuhan Health Commission (No. WX21C09) and the National Natural Science Foundation of China (Nos. 81801157, 81822015, and 81525008).

## Conflict of Interest

The authors declare that the research was conducted in the absence of any commercial or financial relationships that could be construed as a potential conflict of interest.

## Publisher's Note

All claims expressed in this article are solely those of the authors and do not necessarily represent those of their affiliated organizations, or those of the publisher, the editors and the reviewers. Any product that may be evaluated in this article, or claim that may be made by its manufacturer, is not guaranteed or endorsed by the publisher.

## Abbreviations

BAO: basilar artery occlusion
EVT: endovascular treatment
GIH: gastrointestinal hemorrhage
mRS: modified Rankin Scale
NIHSS: National Institutes of Health Stroke Scale Score
pc-ASPECTS: the posterior circulation-Acute Stroke Prognosis Early Computed Tomography Score (pc-ASPECTS).
